# Case report: A rare case of malacoplakia resembling a malignant tumor of the cervix: a case report and review of the literature

**DOI:** 10.3389/fmed.2024.1409239

**Published:** 2024-06-04

**Authors:** Jiaorong Li, Jiaying Mi, Juanjuan Wang, Zhihong Zhuo

**Affiliations:** ^1^Department of Gynecology, Ningbo No. 2 Hospital, Ningbo, Zhejiang, China; ^2^Department of Pathology, Ningbo Clinical Pathology Diagnosis Center, Ningbo, Zhejiang, China

**Keywords:** malacoplakia, malignant tumor of the cervix, pathology, vaginal bleeding, case report

## Abstract

Malacoplakia is a rare chronic granulomatous disease that mostly affects the gastrointestinal tract and urinary tract of immunocompromised patients; malacoplakia rarely effects the female reproductive tract. Here, we report a 56-year-old patient who underwent thymectomy for thymoma and myasthenia gravis prior to developing cervical and vaginal malacoplakia. The patient presented with recurrent vaginal bleeding. We discovered that there were alterations in the cervical cauliflower pattern during colposcopy, which is suggestive of cervical cancer. Pathological examination of the lesion tissue showed that a large number of macrophages aggregated, and M-G bodies with concentric circles and refractive properties were observed between cells. Immunostaining for CD68 and CD163 was positive, and special staining for D-PAS and PAS was positive. The discovery of *Escherichia coli* in bacterial culture can aid in the diagnosis of malacoplakia. Following surgery, we performed vaginal lavage with antibiotics in addition to resection of local cervical and vaginal lesions. This study provides a fresh perspective on the management of genital malacoplakia.

## 1 Introduction

A relatively rare granulomatous condition called malacoplakia primarily impacts the urinary system, but it can also affect the gastrointestinal tract, testicles, prostate, and other organs (1). The causes of malacoplakia and its pathophysiology are unclear. Nonetheless, most studies indicate a strong correlation between infection and the onset of malacoplakia (2). These infections were primarily caused by Acidophilus, Klebsiella species, and *Escherichia coli*. Malacoplakia has also been linked to immunodeficiency, which is thought to be caused by a malfunction in the process of killing intracellular bacteria (3). There is currently little research on malacoplakia in the female vaginal canal. Diagnosing and treating malacoplakia are more difficult for professionals due to its unusual clinical presentation, and clinical misdiagnosis is highly common. The two main treatments for cervical malacoplakia are hysterectomy and antibiotic therapy (2, 4–12).

However, the disadvantages of hysterectomy are obvious, including short-term infections, peripheral organ damage, increased morbidity, and long-term complications such as pelvic organ prolapse and urinary incontinence (13). At present, the treatment and prognosis of malacoplakia patients with uterine preservation are unclear. The cases and treatments reported below provide new ideas for the clinical treatment of genital tract malacoplakia (14).

## 2 Case reports

**Figure 1:**
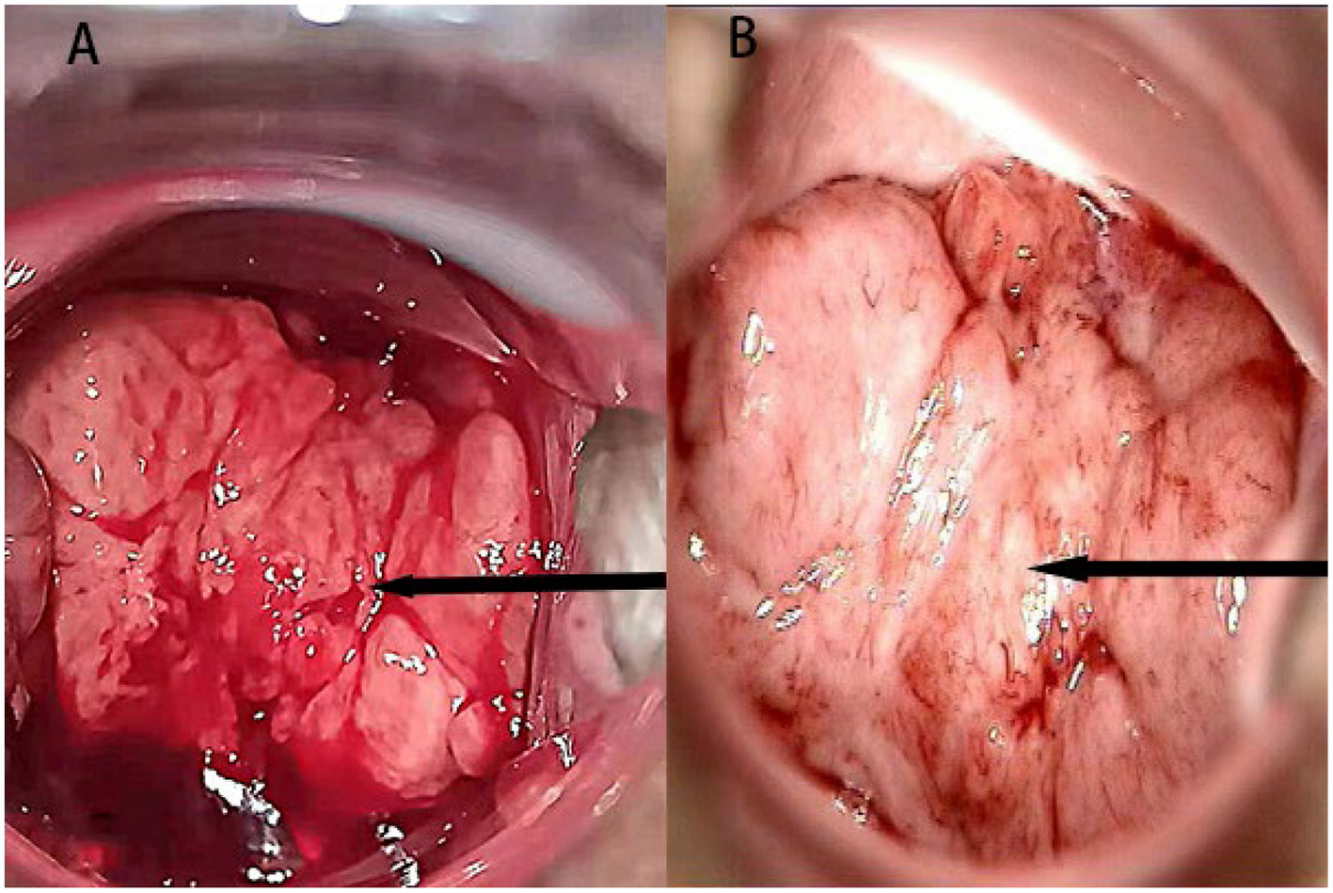
**(A)** A colposcopic image at the time of initial diagnosis; the arrow points to the lesion. **(B)** Review of the colposcopic image in the second month of treatment; the arrow points to the lesion.

**Figure 2:**
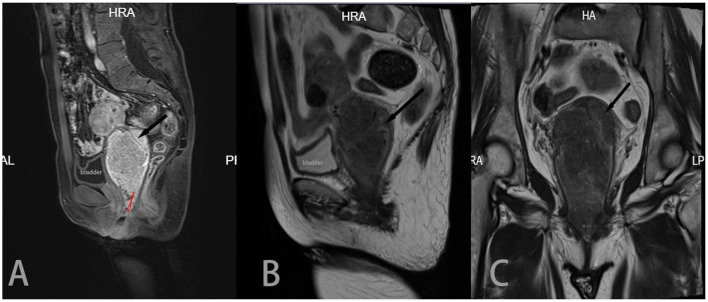
Sagittal T2 **(A)**, sagittal T1 enhancement **(B)** and coronal T2 **(C)** images all show a large cervical mass protruding into the vagina, with a size of approximately 50 * 58 * 91 mm. The mass invaded the cervical interstitium (black arrow), and the surrounding low-signal basal ring was still apparent. In **(B)**, and the lesion appeared in the vagina, occupying 2/3 of the vaginal cavity (red arrow). The uterine body was compressed and moved upward.

**Figure 3:**
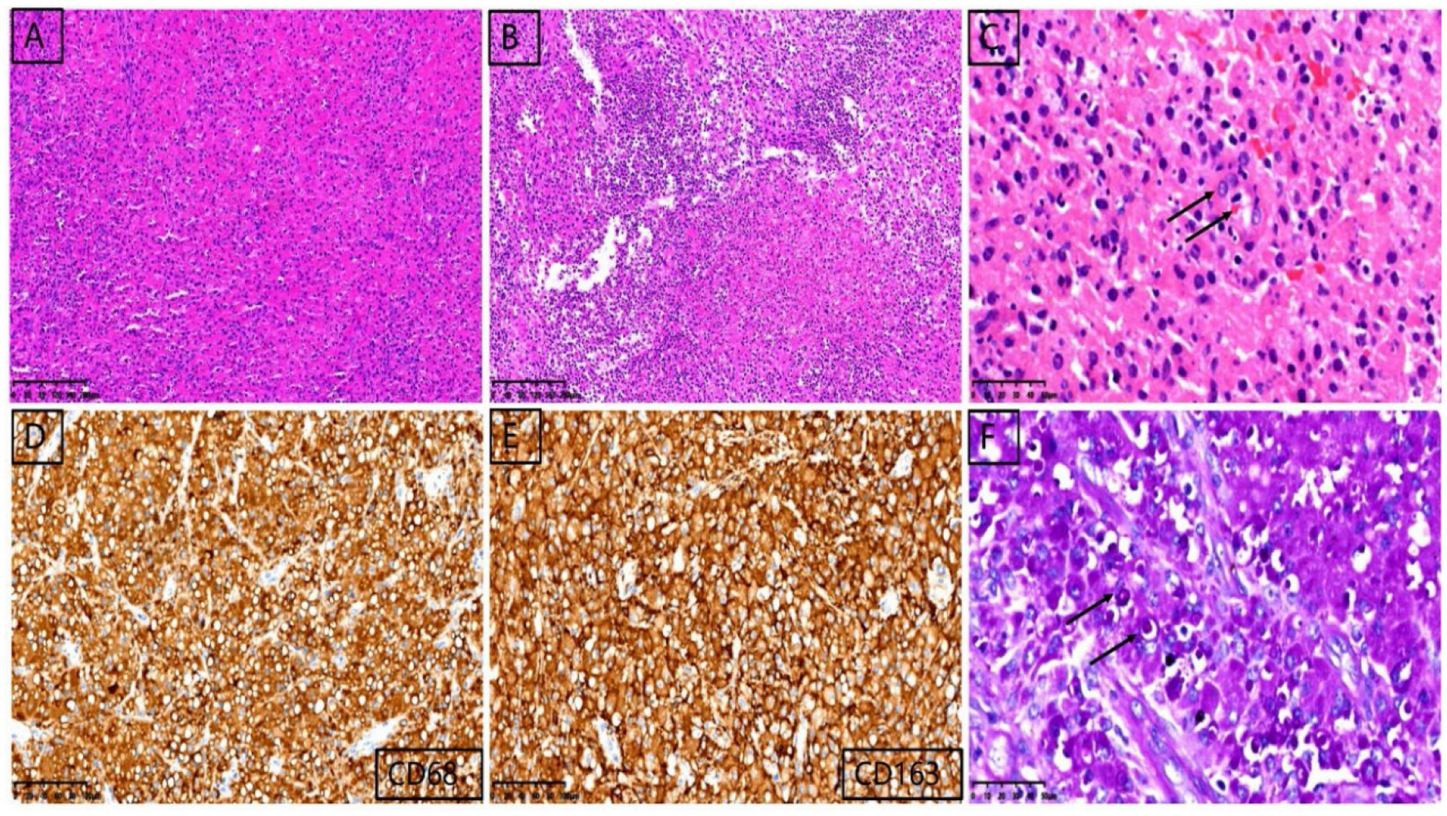
Pathological images of cervical lesions. Microscopy revealed a diffuse inflammatory lesion with a large number of tissue cell clusters [**(A)**, 100X]. In the background of tissue cells, there were varying numbers of plasma cells and lymphocytes, and there may have been bleeding and a small amount of neutrophils [**(B)**, 100X]. Characteristic soft spot bodies (Michaelis Gutmann bodies, MG bodies) were also observed inside and outside the tissue cells. Soft spot bodies were round or oval in shape, with clear boundaries, refractive, alkaline homogeneous shapes, or ring-like structures resembling “owl's eyes” [**(C)**, 400X]. MG bodies are formed by incomplete degradation of bacterial calcification. Immunohistochemistry revealed CD68- and CD163-positive tissue cells [**(D, E)**, 200X), while PAS staining revealed purplish red soft macular bodies [**(F)**, 400X].

At the beginning of 2021, a 56-year-old Chinese woman who complained of recurrent vaginal bleeding for one week visited our hospital. The patient had previously undergone thymectomy due to myasthenia gravis combined with type B2 thymoma, and she had no history of diabetes, AIDS, tuberculosis, etc. Gynecological examination of the patient at the time of treatment revealed that the 5 ^*^ 5 cm cauliflower-like mass on the anterior lip of the cervix involved the vault and easily bled when touched. A gynecologic ultrasound revealed that the cervix was enlarged with solid tumor formation. The outpatient department was highly suspicious of a cervical malignant tumor, so further colposcopy (Figure 1A) and tissue biopsy were performed. Colposcopy revealed that the cervical surface showed cauliflower-like changes, involving the upper 1/3 of the posterior vaginal wall, the upper 1/3 of the left vaginal wall, the front segment of the right vaginal wall, and the front 1/2 of the vaginal wall. No obvious white epithelium was observed, and the iodine test was negative. The microscope showed that a large number of macrophages (tissue cells) aggregated, and concentric and refractive small bodies, called MG bodies, were seen between tissue cells. They can be seen inside or outside the cytoplasm of macrophages (tissue cells) and are characteristic for diagnosing soft spot disease. Immunohistochemical staining for CD68 and CD163 was used to identify tissue cells, while D-PAS and PAS were used to visualize MG bodies. The discovery of *Escherichia coli* in bacterial culture can aid in the diagnosis of soft spot disease. Antibiotics be combined with surgical hysterectomy based on the results of drug sensitivity tests. Due to religious beliefs, the patient refused uterine removal, so she was given 0.2 g intravenous amikacin once a day according to the drug sensitivity test. After one month of treatment, the vaginal bleeding of the patient stopped, the colposcopy mass was smaller than before, and the focus was limited to the cervical surface in the second month (Figure 1B). At the end of 2022, after the patient was infected with COVID-19 and had mild COVID-19 pneumonia, vaginal bleeding occurred again. Pelvic magnetic resonance imaging (MRI) revealed a large cervical space occupying the protrusion into the vagina; this space was thought to be a result of malacoplakia, although cervical malignancies were not excluded (Figure 2). After communicating with the patient, the patient asked to keep their uterus, and after signing the informed consent form, cervical lesion resection + vaginal wall lesion resection was performed. Microscopy revealed a diffuse inflammatory lesion with a large number of tissue cell clusters (Figure 3A, 100X). In the background of tissue cells, there were varying numbers of plasma cells and lymphocytes, and there may have been bleeding and a small amount of neutrophils (Figure 3B, 100X). Characteristic soft spot bodies (Michaelis Gutmann bodies, MG bodies) were also observed inside and outside the tissue cells. Soft spot bodies were round or oval in shape, with clear boundaries, refractive, alkaline homogeneous shapes, or ring-like structures resembling “owl's eyes” (Figure 3C, 400X). MG bodies are formed by incomplete degradation of bacterial calcification. Immunohistochemistry revealed CD68- and CD163-positive tissue cells (Figures 3D, E, 200X), while PAS staining revealed purplish red soft macular bodies (Figure 3F, 400X). After surgery, the method of antibiotic administration was changed, and tobramycin/dexamethasone eye ointment + 0.2 g amikacin were mixed with local vaginal lavage. More than one year after surgery, the disease is well controlled, and there has been no recurrence.

## 3 Discussion

Malacoplakia has been recognized since before 1900 as a very rare disease that is characterized by defects in the mononuclear phagocyte system (15). Malacoplakia related to the female reproductive system is rare. Malacoplakia is usually associated with an immunosuppressive state, indicating that immunity plays an important role in its pathogenesis. Different manifestations of malacoplakia include malignant tumors, SOT, human immunodeficiency virus (HIV), and autoimmune diseases (16–18).

### 3.1 Demographic patterns of malacoplakia of the cervix: age and racial disparities

A previous study revealed that malacoplakia had a high prevalence in the southwestern United States, with patients ranging in age from as young as 6 weeks to as old as 85 years of age (19), usually with the highest prevalence in patients >50 years of age (20). The most recent case of cervical malacoplakia is reported in this paper. To date, a total of 14 cases of cervical malacoplakia have been reported, including that in the present paper, among which the youngest woman was only 27 years old, the oldest patient was 83 years old, and there were 11 cases in women over 50 years old, accounting for 78.57% of all cases; these ages are close to the ages of onset of other malacoplakia diseases (2, 4–12).

### 3.2 Pathogenesis of malacoplakia of the cervix

The etiology and pathogenesis of cervical malacoplakia are still unclear, and the main underlying mechanisms are various microbial infections and immune dysfunction (21). In this case, *Escherichia coli* was found in the vaginal secretions, so we believe that microbial infection may be one of the important factors involved in the pathogenesis of cervical malacoplakia. Moreover, the occurrence of malacoplakia is related to functional defects in macrophages, which block the degradation of phagocytic bacteria by lysosomes, resulting in excessive undigested bacterial debris in the cytoplasm (22–24). Our patient had previously undergone a thymectomy for myasthenia gravis with type B2 thymoma. Thymoma-associated myasthenia gravis is a paraneoplastic disease, and myasthenia gravis is the most widely reported autoimmune disease associated with thymoma (25). There is evidence that cholinergic receptor agonists, such as chlormethine, combined with antibiotics may improve the function of macrophages by correcting lysosomal defects. Therefore, we speculate that cholinergic receptor antibodies in malacoplakia patients may affect the function of macrophages, thus driving the phagocytosis of pathogenic bacteria in patients (26). Therefore, we hypothesized that the patient's previous history of myasthenia gravis combined with thymoma may have induced the development of malacoplakia.

### 3.3 Challenges in the clinical diagnosis of malacoplakia of the cervix: overlapping symptoms

Most patients with malacoplakia present with abnormal vaginal bleeding and ulcerative changes in the cervix, which can be seen with the naked eye (27). The similarity of clinical manifestations often leads us to overlook cervical malacoplakia and misdiagnose it as a malignant tumor of the cervix (28). Our patient saw a doctor due to abnormal vaginal bleeding. During gynecological examination and colposcopy, a cauliflower-like cervical tumor was found. Ultrasound revealed that the cervix was enlarged with a solid tumor-like appearance. Because cervical cancer was suspected, we conducted a colposcopy examination and found that the lesion was soft, yellow, slightly raised, and fused into a 5 ^*^ 5 cm cauliflower-like plaque. A histological biopsy was taken. However, to our surprise, the pathological report suggested that this was a case of malacoplakia. Here, we emphasize the clinical significance of malacoplakia in the differential diagnosis of gynecological diseases. Malacoplakia may mimic malignant tumors, which can be a challenge for obstetricians and gynecologists.

### 3.4 Identification of malacoplakia in the genital tract

To date, there have been fewer than 40 reported cases of female genital malacoplakia, 14 of which were cervical malacoplakia (including this case); most of these patients presented with vaginal and endometrial soft spots, and ovarian and fallopian tube invasion was rarer (29). Malacoplakia involving the cervix, endometrium and vagina has similar clinical manifestations, mainly abnormal uterine bleeding, postmenopausal vaginal bleeding and increased secretions (30). The ultrasound characteristics of endometrial malacoplakia include anechoic fluid expansion in the endometrial cavity in the acute stage, irregular and heterogeneous thickening, and endometrial hypopogenicity in the chronic stage; gynecological examinations generally have no specific findings (31). The imaging characteristics of cervical malacoplakia include a hypoechoic space in the cervix. Cervical lesions can be detected via gynecological examination and confirmed via cervical lesion biopsy. Vaginal malacoplakia can be detected through gynecological examination, vaginal lesions, and biopsy, and abnormalities can be detected in the uterus and cervix (7). Ovarian and tubal malacoplakia often manifest as abdominal pain and abdominal discomfort before surgery, and imaging can reveal space in the accessory area. Operations can show that lesions directly spread and invade the surrounding tissues, similar to tumors, but postoperative pathology will suggest malacoplakia (32).

### 3.5 Pathological features of malacoplakia of the cervix

Malacoplakia is a chronic granulomatous disease that is characterized by a large number of tissue cells that are visible under the microscope, with a background of small lymphocytes, plasma cells, and neutrophils (26). Among them, circular or oval shaped cells are observed, with clear boundaries, refraction, alkaline homogeneity, or a ring-like structure resembling “owl's eyes”. Immunohistochemically, there are more CD68 and CD163 positive tissue cells, and unstained circular or oval vacuolar structures can be seen inside tissue cells (29). The MG bodies are specifically stained with D-PAS. PAS can mark MG bodies, which are purple–red in color. The diagnosis is consistent with soft spot disease. Due to the rarity of this disease, its clinical symptoms and general features are nonspecific, and there is a lack of sufficient understanding. Thus, misdiagnosing this disease as a malignant tumor, especially using frozen specimens obtained during surgery, is easy, and misdiagnosis and missed diagnoses can occur. Therefore, differential diagnosis is necessary. (1) The differential diagnosis for endometrial poorly differentiated carcinoma is as follows: when malacoplakia occurs in the uterine cavity, it often manifests as vaginal bleeding, menstrual changes, ultrasound detection of a space occupying the uterine cavity, and microscopic masses of tissue cells that are easily mistaken for epithelial cells. Especially during intraoperative frozen sectioning, due to the lack of fixed tissue and atypical cell morphology, malacoplakia can be misdiagnosed as poorly differentiated endometrial carcinoma. In endometrial cancer, CK (AE1/AE3) and vimentin are positive, while CD68 is negative, and the Ki67 proliferation index is significantly greater than that in malacoplakia (33, 34). (2) The differential diagnosis for malignant melanoma is as follows: when malacoplakia is accompanied by bleeding, the lesion appears dark brown. Under a microscope, tissue cells are prone to morphology similar to that of malignant melanoma cells. Malignant melanoma cells exhibit obvious atypia, with large purple–red nucleoli visible and melanin visible in the cytoplasm. The immunohistochemical markers HMB-45, Melan-A, and S-100 are positive (35). (3) Xanthogranulomatous and histiocytic endometritis are commonly observed in postmenopausal women and are characterized by vaginal bleeding or fluid flow, often accompanied by cervical stenosis or pyometra with generally brownish yellow brittle tissue. Microscopically, patients with xanthogranulomatous and histiocytic endometritis show a large number of tissue cells with eosinophilic or foam-like cytoplasm. The cytoplasm is also rich in lipids or hemosiderin. There are also plasma cells, lymphocytes and neutrophils in the background (36, 37). Unlike malacoplakia, these patients lack characteristic MG bodies. This disease can be distinguished based on medical history.

### 3.6 Individualized management of malacoplakia of the cervix

Currently, there are no definitive guidelines for the treatment of malacoplakia. The main therapeutic approaches are antimicrobial therapy, a reduction in the use of immunosuppressive drugs and surgical treatment (23, 24). Quinolone antimicrobials (methotrexate, ciprofloxacin) have good cell membrane penetration and are therapeutically effective. However, quinolones block neuromuscular transmission, and there is a possibility of myasthenia gravis (38, 39). Early antimicrobial treatment before malacoplakia causes severe and extensive pathological damage can prevent this pathological damage (40). In this case, the patient was sensitive to aminoglycoside antibiotics, so we administered amikacin and tobramycin ointment for anti-infection treatment. In addition, another treatment strategy is immunotherapy. Cholinergic receptor agonists and vitamin C can alleviate immune dysfunction. Ascorbic acid can enhance lysozyme damage caused by immune deficiency. Therefore, the combination of antibiotics, vitamin C, and cholinergic drugs may have a certain effect (41–44).

Surgery may be recommended when conventional drug therapy fails (2, 4–12). Thirteen cases of cervical malacoplakia have been reported; three patients were treated with antibiotics, five were treated with hysterectomy, and the remaining five were either not treated or died before treatment. Hysterectomy was performed in all surgically treated patients, which may be related to the difficulty of distinguishing cervical malacoplakia from cervical malignancy (29) (Table 1). Due to differences in culture and religious beliefs, most Chinese women want to preserve the uterus. For our patient, we intravenously administered antibiotics they were sensitive to immediately after diagnosis. In the first month, the cervical space occupation tended to decrease. However, after infection with COVID-19, the cervical space occupied became larger. This effect may be related to COVID-19 attacking the immune system (45, 46). Due to the special anatomical properties of the cervix, we first performed a colposcopic biopsy before the operation, and the pathology confirmed cervical malacoplakia. Therefore, we developed a personalized operation involving the resection of cervical lesions and vaginal wall lesions. We also administered a special vaginal lavage after surgery. The optimal duration of antibiotic therapy for patients with malacoplakia is unclear, and typically ranges from 12 weeks to 6 months (14). We chose to administer the drug vaginally for 3 months continuously, and the patient did not relapse within 12 months after surgery.

**Table 1:** Summary of the previous cases reported of malacoplakia of the uterine cervix in literature along with our case.

**Age**	**Clinical presentation**	**Initial diagnosis**	**Anamnesis**	**Treatment**	**Follow-up**	**Reference**
64	Reproductive tract bleeding	Not described	Transitional cell carcinoma of the urinary bladder. Nulligravid. Use prednisone	Antibiotic treatment after a cervical biopsy	The vaginal vault recurred 2 years after surgery.	(7)
71	Reproductive tract bleeding; abdominal pain	Not described	Cholecystectomy	Hysterectomy Electrocautery of vaginal lesion	No recurrence	(5)
83	Reproductive tract bleeding	Not described	Xanthogranulomatous pyelonephritis	Hysterectomy	The vaginal stump recurred 14 months after surgery.	(11)
69	Uterine prolapse Cervical ulceration	Uterine prolapse. Proctopto-ma	Uterine prolapse	Hysterectomy. Partial resection of the vagina	Not reported	(27)
60	Reproductive tract bleeding	Therioma	Rheumatoid. Use cortisol	Not reported	Not reported	(4)
74	Reproductive tract bleeding	Therioma	No	Antibiotic	Not reported	(8)
27	Reproductive tract bleeding Cervical ulceration	Cervical malignancy	AIDS(Acquired immune deficiency syndrome)	Antibiotic	Follow-up failure	(2)
36	Reproductive tract bleeding Cervical ulceration	Cervical malignancy	AIDS(Acquired immune deficiency syndrome)	Died before treatment	Died before treatment	(2)
81	Reproductive tract bleeding	Cervical malignancy	Nulligravid	Not reported	Not reported	(12)
78	Reproductive tract bleeding. Abdominal pain	Cervical malignancy	Sjögren's syndrome. Use cortisol.	Hysterectomy	There was no recurrence at 13 months after surgery.C	(29)
72	Reproductive tract bleeding	Cervical malignancy	Use cortisol	Died before treatment	Died before treatment	(9)
66	Reproductive tract bleeding	Cervical malignancy	No	Antibiotic	Not reported	(9)
78	Reproductive tract bleeding	Not described	No	Antibiotic	Not reported	(10)
56	Reproductive tract bleeding	Cervical malignancy	Myasthenia gravis. Thymoma	Resection of the cervical and vaginal lesions	There was no recurrence at 12 months after surgery.	Present case

## 4 Conclusion

Malacoplakia is a rare systemic disease that is usually seen in immunocompromised patients, and the common treatment regimen is intravenous or oral antibiotics combined with total hysterectomy. In our case, we used the first treatment protocol involving antibiotic vaginal lavage after combined resection of cervical and vaginal lesions, which was a new approach for the treatment of cervical malacoplakia. Early and accurate diagnosis and individualized treatment are the basis for improving patient prognosis, and we hope that in future studies, we can explore the etiology, pathogenesis, imaging characteristics, and treatment modalities of cervical malacoplakia in greater depth to improve the quality of life of patients and the cure rate of this disease.

## Data availability statement

The raw data supporting the conclusions of this article will be made available by the authors, without undue reservation.

## Ethics statement

The studies involving humans were approved by Ethics Committee of Ningbo No.2 Hospital. The studies were conducted in accordance with the local legislation and institutional requirements. The participants provided their written informed consent to participate in this study. Written informed consent was obtained from the individual(s) for the publication of any potentially identifiable images or data included in this article.

## Author contributions

JL: Writing – original draft, Writing – review & editing. JM: Software, Writing – original draft, Writing – review & editing. JW: Data curation, Writing – review & editing. ZZ: Funding acquisition, Resources, Writing – review & editing.
